# How does apolipoprotein E genotype influence the relationship between physical activity and Alzheimer’s disease risk? A novel integrative model

**DOI:** 10.1186/s13195-023-01170-4

**Published:** 2023-01-27

**Authors:** Jaisalmer de Frutos Lucas, Kelsey R. Sewell, Alejandra García-Colomo, Shaun Markovic, Kirk I. Erickson, Belinda M. Brown

**Affiliations:** 1 Experimental Psychology, Cognitive Processes and Logopedia Department, School of Psychology, Universidad Complutense de Madrid, 28223 Pozuelo de Alarcón, Spain; 2 Centre for Precision Health, Edith Cowan University, Joondalup, Western Australia 6027 Australia; 3 Departamento de PsicologíaFacultad de Ciencias de la Vida y de la Naturaleza, Universidad Antonio de Nebrija, 28015 Madrid, Spain; 4 Centre for Healthy Ageing, Health Futures Institute, Murdoch University, Murdoch, Western Australia 6150 Australia; 5 Australian Alzheimer’s Research Foundation, Sarich Neuroscience Research Institute, Nedlands, Western Australia 6009 Australia; 6 Department of Psychology, University of Pittsburgh, Pittsburgh, PA 15260 USA; 7 PROFITH “PROmoting FITness and Health Through Physical Activity” Research Group, Sport and Health University Research Institute (iMUDS), Department of Physical and Sports Education, Faculty of Sport Sciences, University of Granada, 18071 Granada, Spain; 8 AdventHealth Research Institute, Orlando, FL 32804 USA; 9 School of Medical and Health Sciences, Edith Cowan University, Joondalup, Western Australia 6027 Australia

**Keywords:** *APOE ε4*, Alzheimer’s disease, Physical activity, Amyloid pathology, Tau pathology, Cerebrovascular health, Neurotrophic factors, Neuroinflammation, Glucose metabolism, Mitochondrial dysfunction

## Abstract

**Background:**

Wide evidence suggests that physical activity (PA) confers protection against Alzheimer’s disease (AD). On the other hand, the apolipoprotein E gene (*APOE*) ε4 allele represents the greatest genetic risk factor for developing AD. Extensive research has been conducted to determine whether frequent PA can mitigate the increased AD risk associated with *APOE* ε4. However, thus far, these attempts have produced inconclusive results. In this context, one possible explanation could be that the influence of the combined effect of PA and *APOE ε4* carriage might be dependent on the specific outcome measure utilised.

Main body.

In order to bridge these discrepancies, the aim of this theoretical article is to propose a novel model on the interactive effects of PA and *APOE* ε4 carriage on well-established mechanisms underlying AD. Available literature was searched to investigate how PA and *APOE* ε4 carriage, independently and in combination, may alter several molecular pathways involved in AD pathogenesis. The reviewed mechanisms include amyloid beta (Aβ) and tau deposition and clearance, neuronal resilience and neurogenesis, lipid function and cerebrovascular alterations, brain immune response and glucose metabolism. Finally, combining all this information, we have built an integrative model, which includes evidence-based and theoretical synergistic interactions across mechanisms. Moreover, we have identified key knowledge gaps in the literature, providing a list of testable hypotheses that future studies need to address.

**Conclusions:**

We conclude that PA influences a wide array of molecular targets involved in AD neuropathology. A deeper understanding of where, when and, most importantly, how PA decreases AD risk even in the presence of the *APOE* ε4 allele will enable the creation of new protocols using exercise along pharmaceuticals in combined therapeutic approaches.

## Key points

- Physical activity (PA) and *APOE* ε4 contribute to AD risk in opposite directions.
- The combined effect of PA and *APOE* ε4 varies across several mechanisms in AD.
- We propose an integrative model of how PA might partially offset *APOE* ε4 damage.
- This integrative model could aid to formulate new combined therapeutic strategies.

## Background and objective

Physical activity (PA) engagement is one of the most effective methods for reducing the risk of multiple diseases, including cancer, dementia and cardiovascular disease [1]. One example of the potential neural benefits of PA engagement is the slowing of neuropathological processes associated with Alzheimer’s disease (AD). AD is a multifactorial disorder, where numerous modifiable and non-modifiable elements contribute to increased disease risk. One of the most relevant non-modifiable factors that contributes to AD risk is the carriage of the apolipoprotein E (*APOE*) ε4 gene allele. Hence, one relevant question emerges: Does the *APOE* genotype influence the relationship between PA and AD risk? In other words, can a modifiable factor (i.e. PA) mitigate some of the risks of a non-modifiable factor (*APOE* ε4 carriage)?

PA is defined as any bodily movement that raises energy expenditure above basal consumption and can be differentiated from exercise which is a planned, structured and repetitive type of PA that serves a specific goal [1, 2]. Throughout this article, we will preferably use the term PA unless the specific type of PA involved meets the criteria for exercise. PA has been associated with decreased amyloid and tau pathology, preserved brain structure (particularly of brain areas more vulnerable to AD), improved cognitive outcomes and overall reductions in AD incidence [3–6]. The beneficial effects of PA on the brain are likely mediated via its influence on multiple systems, including the immune, cerebrovascular, neuroendocrine and neurotrophic response [7], providing multiple pathways through which PA could contribute to reduced incidence of multiple diseases, including AD.

On the other hand, the *APOE* ε4 allele is present in 60–80% of AD cases and increases AD risk in a dose-dependent manner [8]. The number of ε4 alleles has also been found to be negatively associated with age at onset [9]. Apolipoprotein E (ApoE—protein) is the most abundant apolipoprotein in the brain, where it plays a fundamental role in cholesterol and lipid transport and metabolism. The *APOE* polymorphisms can substantially change the structure and function of the protein, modifying its binding properties. In a recent publication, Flowers and Rebeck [10] have summarised the main differences in structure and neural effects between the three most common isoforms. According to the authors, the degree of lipidation of the three common ApoE isoforms varies, where ApoE2 is associated with more lipidated particles and ApoE4 with less lipidated particles than ApoE3 (ApoE2 > ApoE3 > ApoE4). The level of lipidation of ApoE affects the efficiency of lipid transport and receptor-binding interaction. ApoE4 binding to high-density lipoproteins (HDL) is lower compared with other isoforms, which makes the aggregation of unlipidated ApoE monomers more likely. These large aggregates are more toxic to neurons than those formed by ApoE2 and ApoE3.

Here, we intend to detangle how PA and *APOE* genotype interact in their contribution to AD risk as a complex disease. Over the last two decades, scientists have attempted to answer this question, yielding vastly inconsistent results [11, 12]. In a recent systematic review [12], we identified, along with multiple methodological issues, one critical consideration that could partially explain the lack of convergent results within this field: The influence of the combined effect of PA and *APOE* ε4 carriage might be dependent on the specific outcome measure used. Studies examining various AD-related traits (e.g. amyloid beta burden or cortical atrophy) might show diverse results because the biological pathways involved are differentially influenced by PA, *APOE* ε4 carriage and their interaction.

The aim of this theoretical article is to propose a novel model of how PA and *APOE* ε4 carriage, independently and in combination, may alter well-established mechanisms underlying AD pathogenesis. The reviewed mechanisms include amyloid beta (Aβ) and tau deposition and clearance, neuronal resilience and neurogenesis, lipid function and cerebrovascular alterations, brain immune response and glucose metabolism. The list of mechanisms addressed has been primarily ordered based on their involvement in AD pathology and only secondarily on the amount of evidence available regarding how they are affected by PA and *APOE* genotype. Moreover, our integrative model includes evidence-based and theoretical synergistic interactions across mechanisms, as well as a proposal of testable hypotheses that future studies need to address. A better understanding of where, when and most importantly, how, PA decreases AD risk in the presence of the *APOE* ε4 allele is essential to formulate combined therapeutic approaches in the absence of a cure for AD.

## How do *APOE* genotype and physical activity influence Alzheimer’s neuropathology?

### Amyloid pathology

Aggregation of Aβ and the resultant plaque formation is one hallmark of AD, initiating a series of pathological cascades leading to neuronal death and cognitive decline [13]. An intermediary of Aβ deposition, soluble Aβ oligomers, is the most neurotoxic aggregates and is associated with neural dysfunction, induce neuronal apoptosis, and inhibition of synaptic long-term potentiation (LTP) [14]. As summarised by Huang and Liu [15], Aβ oligomers contribute to the neurotoxic environment through receptor binding, mitochondrial dysfunction and tau pathologies, resulting in declines in cognitive function. *APOE* ε4 carriers show increased levels of soluble Aβ compared to non-carriers, detailing the central role of apoE Aβ metabolism [16], and exercise has also been shown to reduce levels of soluble Aβ [17].

In their review, Brown et al. concluded that in animal models, both exercise and PA (forced and voluntary wheel running, respectively) are associated with lower levels of cortical Aβ [11]. Importantly, exercise may contribute to both reduced production and increased clearance of cortical Aβ. Facilitation of Aβ accumulation may occur via processing of amyloid precursor protein (APP), which is processed either via the non-amyloidogenic pathway (leads to neuronal growth and excitability) or amyloidogenic pathway (produces the building blocks for Aβ plaques) [20]. Exercise can modulate enzymes which are involved in APP cleavage, such as ADAM-10 [21], presenilin (PS1) [22] and BACE1 [23], reducing APP cleavage via the amyloidogenic pathway, and thereby decreasing the production of Aβ [24] in mouse models of AD.

ApoE4 plays a central role in driving Aβ accumulation, through both facilitating Aβ aggregation and inhibiting Aβ clearance [25]. For example, ApoE4 is less efficient at clearing soluble Aβ from the interstitial fluid (ISF), as opposed to ApoE2 or ApoE3 [26]. ApoE isoforms may also mediate the clearance of Aβ via the blood–brain barrier (BBB), with ApoE4 being the least efficient [27]. Indeed, a combination of chronically elevated IL-6 (a pro-inflammatory cytokine) and BBB dysfunction has been associated with greater Aβ in *APOE* ε4 carriers only [28]. Additionally, ApoE4 binding to Aβ may alter the Aβ clearance pathway from the LDL receptor–related protein 1 (LRP1) to the VLDL receptor (VLDLR), which internalises Aβ-ApoE4 complexes at the BBB more slowly than LRP1 [27]. However, ApoE may also compete with Aβ for cellular uptake via LDLR receptors [29]. It remains unclear whether ApoE facilitates cellular Aβ uptake via forming Aβ-ApoE4 complexes, whose clearance efficiency is ApoE isoform dependent, or whether ApoE may compete with Aβ for receptor binding [30]. Finally, the ApoE4 isoform may be less efficient at promoting Aβ degradation via neprilysin (an Aβ degrading enzyme), compared to ApoE2 and ApoE3 [31].

**Fig. 1:**
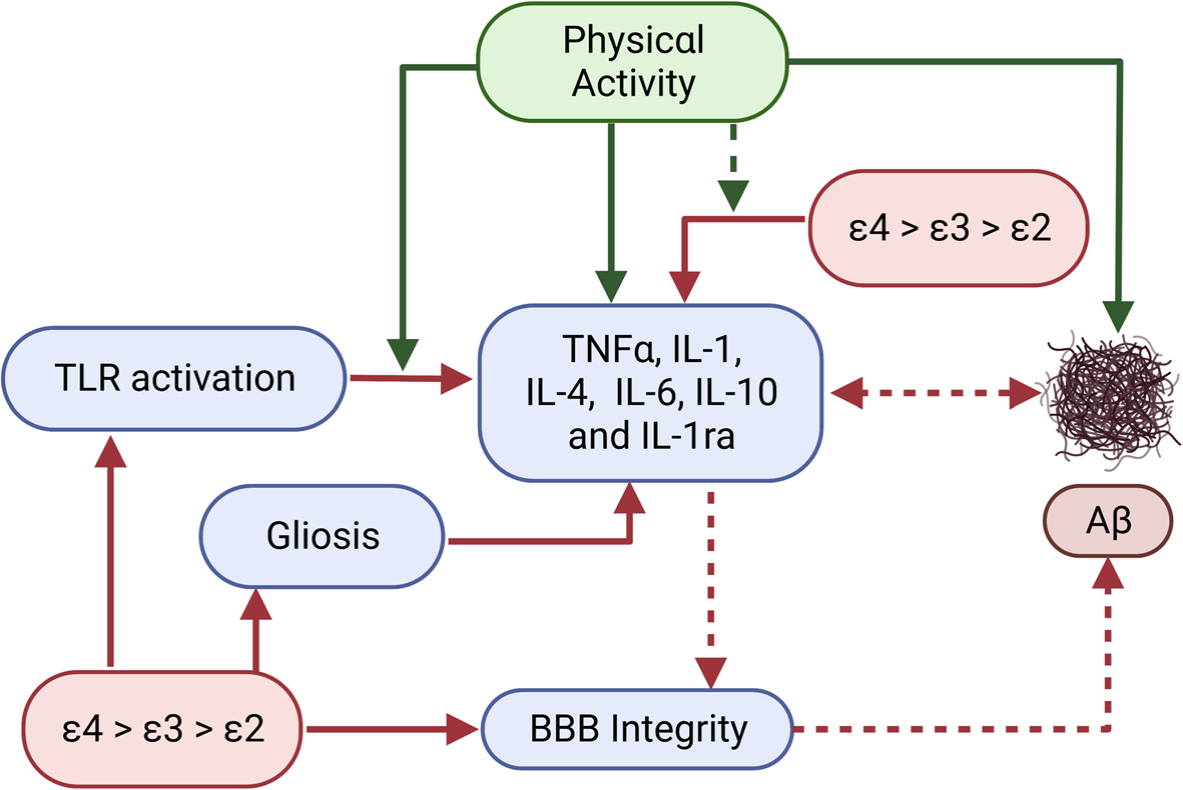
Hypothesised associations between apolipoprotein E gene allele, exercise, inflammatory factors and amyloid-beta. Solid lines indicate empirically supported pathways, and dashed lines indicate hypothesised pathways with preliminary evidence. Green lines indicate a positive effect, and red lines indicate a negative effect. Abbreviations: Aβ amyloid-beta, *APOE* apolipoprotein E, BBB blood–brain barrier, IL interleukin, TLR toll-like receptor, TNF tumour necrosis factor. Created by KS with BioRender.com

Current evidence from human research indicates PA-induced reductions in brain Aβ may be greater for *APOE ε4* allele carriers, compared to *ε4* non-carriers [12]. Although this evidence is relatively consistent, there is very little research examining the potential mechanisms for this interaction. However, when investigated separately, ApoE and PA have shared mechanistic pathways to influence AD biomarkers; thus, it is likely that there is an interaction between these factors on the molecular level. For example, ApoE may affect the clearance of soluble Aβ in the ISF in an isoform-dependent manner (ApoE4 < ApoE3 ≤ ApoE2). However, exercise can accelerate the movement of ISF drainage fluids, accelerating Aβ clearance and reducing Aβ accumulation [32]. Theoretically, through this mechanistic pathway, PA could attenuate some of the negative impacts of the ε4 allele, which is consistent with studies which show greater exercise-induced benefit for ε4 carriers. Moreover, ApoE and exercise both act to regulate proteases such as LRP1 and neprilysin, which may secondarily influence Aβ degradation and clearance. For example, PA may upregulate LRP1, leading to increased Aβ clearance [33]. However, the effectiveness of this pathway may be ApoE isoform dependent, in that Aβ binding to ApoE4 alters the clearance pathway from LRP1 to VLDL, which is a less efficient clearance method [27]. Thus, if ApoE4 is altering this clearance pathway, the exercise-induced increase in levels of LRP1 may be less effective for increasing Aβ degradation. Additionally, exercise has been shown to upregulate neprilysin and insulin-degrading enzyme (IDE), leading to increased Aβ degradation in animal models [33]. Post-mortem studies show ε4 carriers have reduced expression of neprilysin and IDE in the brain, compared to ε4 non-carriers, and efficiency of Aβ degradation via neprilysin may be ApoE isoform dependent (ApoE4 being the least efficient) [31, 34, 35]. Thus, exercise-induced increases in neprilysin and IDE could partially mitigate Aβ degradation inefficiency in ε4 carriers specifically, but further research is needed to test this hypothesis. Figure 1 presents a summary of hypothesised associations between *APOE* gene allele carriage, physical activity, inflammatory factors and Aβ.

### Tau pathology

Neurofibrillary tangles (NFTs) are a second hallmark of AD (additional to Aβ plaques) and are composed of hyperphosphorylated or abnormally phosphorylated tau aggregates [36]. Importantly, tau aggregation is associated with clinical symptom onset and cognitive function in preclinical AD [37]. Animal models of AD and other tauopathies show that exercise and PA can reduce hippocampal tau pathology and tau phosphorylation [38]. Two main tau kinases (glycogen synthase kinase 3 (GSK3) and cyclin-dependent kinase 5 (CDK5)), important for tau phosphorylation, may be mechanisms through which PA reduces brain (hyper)phosphorylated tau [18]. However, the mechanistic link between PA and tau is poorly understood, with one animal study showing that GSK3, but not CDK5, plays a mediating role in the relationship between exercise and tau phosphorylation, and other studies showing no effect of PA on various tau kinases [22, 38]. Animal models suggest that upregulation of pro-inflammatory cytokines increases tau hyperphosphorylation, and higher PA levels in humans are associated with lower CSF tau and IL-8 [39, 40].

ApoE4 increases tau hyperphosphorylation; however, it is currently unclear whether this relationship is dependent on the presence of Aβ [41, 42]. Indeed, a recent study showed that ApoE may facilitate tau phosphorylation induced by Aβ oligomers in an isoform-dependent manner, with ApoE4 being the most potent [43]. Elevated CSF tau levels have been associated with decreased cortical plasticity and cognitive decline in *APOE* ε4, but not *APOE* ε3 carriers [44], supporting the notion that ApoE4 may enhance tau-mediated neurodegeneration [42].

There is very limited evidence for how ApoE and PA may interact to influence tau pathology. However, a recent study [45] showed that overexpression of LDLR in tau transgenic mice reduces brain ApoE and attenuates tau pathology and neurodegeneration. As detailed above, PA may upregulate LDLR, thus indicating a potential mechanistic pathway through which PA and ApoE may interact to influence tau pathology; however, further research is required. Additionally, because the accumulation of tau pathology may be Aβ-dependent, future research should consider the role of Aβ in this process.

### Neurotrophic factors

An integral component of PA-promoted neuroprotection is the proliferation of neurotrophins, which are a group of endogenous proteins critical for neuronal survival, regeneration and growth [46]. In the context of AD, optimal neurotropic functioning might be key to counterbalancing structural damage through synaptic plasticity. Moreover, neurotrophic dysregulation has been reported early in the disease [47]. Prominent families of neurotrophins include brain-derived neurotrophic factor (BDNF), insulin-like growth factor (IGF) and vascular endothelial growth factor (VEGF). BDNF is found in high concentrations in the hippocampus and is integral to long-term potentiation, memory formation and synaptic function [48]. Upregulation of BDNF subsequently promotes neurogenesis and cell formation and leads to downstream cognitive benefits [49]. Insulin-like growth factor 1 (IGF-1) is a growth hormone which is critical to cellular development (anabolism) and maintenance in the CNS, glucose metabolism and insulin regulation [50]. VEGF is also a key promotor of hippocampal angiogenesis and microvasculature formation [51]. Critically, animal studies have shown that increased IGF-1 expression via exercise also facilitates both blood vessel proliferation [52] and hippocampal neurogenesis [50].

A single bout of aerobic or resistance exercise can result in discernible increases in peripherally circulating BDNF (which is also a myokine), both in healthy older adults and those exhibiting cognitive decline [53, 54]. Although factors such as exercise session length and intensity likely determine the volume of BDNF expression increase [54]. Studies of both voluntary and forced wheel running in rodents have found that aerobic exercise is effective in upregulating hippocampal BDNF, tropomyosin receptor kinase B (TrkB, a BDNF receptor) and VEGF concentrations [49, 55–57]. Additionally, high-intensity exercise, which induces lactic acid build-up, appears to be most effective at stimulating VEGF hippocampal expression and angiogenesis [56]. PA-induced increases in neurotrophic concentrations and the resulting neurogenesis can promote cognitive improvements and increases in hippocampal volume in humans [58]. Moreover, serum IGF-1 concentration is promoted by resistance exercise, likely as a result of its anabolic action and role in muscle growth [59, 60].

The presence or absence of the *APOE* ε4 allele may play a key role in neurotrophic response to PA and exercise. Serum BDNF levels are lower in *APOE* ε4 AD participants compared to both non-*APOE* ε4 carriers diagnosed with AD and cognitively normal older adults [61, 62]. Furthermore, low serum BDNF could serve as a predictor of conversion from mild cognitive impairment (MCI) to AD in *APOE* ε4 carriers [63]. The variations in BDNF secretion as a function of *APOE* status could occur via multiple pathways, including the direct inhibition of astrocytic expression of BDNF in ε4 carriers [64] and/or epigenetic repression of BDNF expression in neurons [64]. Similarly, Keeney and colleagues [65] reported a novel association between *APOE* genotype and IGF-1, where transgenic mice modified to carry the human *APOE* ε4 allele had reduced cortical IGF-1 protein and hippocampal IGF-1 mRNA, compared to mice carrying the *APOE* ε2 allele. There was little difference in IGF-1 gene expression between ε4 and ε3 mice [65]. Additionally, *APOE* ε4 transgenic mice had a reduced concentration of hippocampal VEGF compared to *APOE* ε3 animals [66].

The interaction between ApoE, PA and neurotrophic factors is likely complex and multi-faceted. Although *APOE* ε4 carriers benefit from PA engagement, ε4 carriage, especially in homozygotes, can diminish neurotrophic function [67], potentially through the detrimental effect of the *APOE* ε4 allele on BDNF secretion and maturation. Exercise-induced BDNF could still support neurogenesis and synaptogenesis in *APOE* ε4 carriers, yet less effectively than in ε3 and ε2 carriers. Animal studies have shown that PA can increase levels of BDNF, its TrkB receptor (reduced by 50% in the presence of ε4 allele) and synaptophysin (a marker of synaptic function) in transgenic ε4 mice, to the level of ε3 mice [68]. Accordingly, PA could mitigate some of the negative effects ε4 allele possession has on BDNF secretion. However, there is also evidence of increased neuronal apoptosis following voluntary wheel running in *APOE* ε4 mice and increased neurogenesis in *APOE* ε3 mice [69]. Although this study did not examine BDNF levels, it does indicate that PA-induced neurotrophic change and the resultant neuronal effects may be ApoE isoform-dependent.

**Fig. 2:**
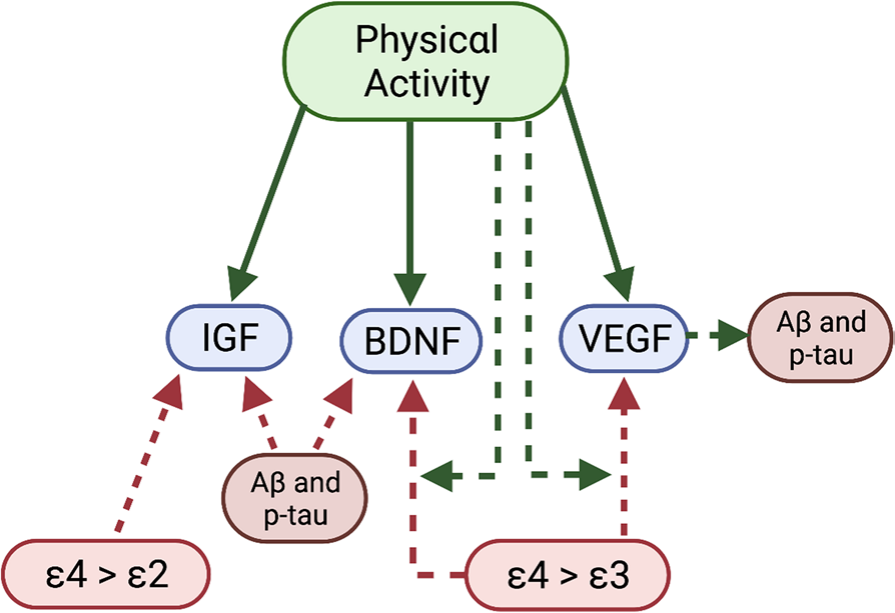
Hypothesised associations between apolipoprotein E gene allele, physical activity and neurotrophic factors. Solid lines indicate empirically supported pathways, and dashed lines indicate hypothesised pathways with preliminary evidence. Green lines indicate a positive effect, and red lines indicate a negative effect. E4 > E2 indicates a greater effect for E4 carriers compared to E2 carriers, and E4 > E3 indicates a greater effect for E4 carriers compared to E3 carriers. Abbreviations: Aβ amyloid-beta, *APOE* apolipoprotein E, BDNF brain-derived neurotrophic factor, IGF insulin-like growth factor, p-tau phosphorylated tau, VEGF vascular endothelial growth factor. Created by KS with BioRender.com

The association between VEGF expression and *APOE* genotype is also poorly understood, owing to diverse findings in the peripheral and central expression of VEGF in AD samples compared with cognitively normal older adults [70]. *APOE* ε4 transgenic mice have a reduced concentration of hippocampal VEGF compared to ε3 animals, while subsequent treatment with intra-hippocampal VEGF-A injections reversed aggregation of Aβ-42 and *p-*tau in *APOE* ε4 mice [66]. Since there is evidence that exercise can stimulate hippocampal VEGF expression [56], it is plausible that PA may ameliorate some of the negative impacts ε4 carriage has on hippocampal VEGF and subsequent aggregation of Aβ-42 and *p-*tau. Higher expression of VEGF and a co-receptor (neuropilin 1) have been associated with poorer cognitive performance in *APOE* ε4 carriers, while the inverse was true for non-carriers, suggesting any VEGF-derived neuroprotection was attenuated by possession of the ε4 allele [70]. However, it remains unclear whether exercise-induced upregulation of VEGF may protect against cognitive decline or neurodegeneration in ε4 carriers. Further research assessing the neurotrophic response to exercise or longer-term PA patterns as a function of *APOE* genotype along with downstream effects on neurocognitive health is warranted. Figure 2 presents a summary of hypothesised associations between *APOE* gene allele carriage, physical activity and neurotrophic factors.

### Cerebrovascular alterations

PA exerts a positive response in the cardiovascular system, which may also benefit the brain. Greater PA engagement is associated with increased cerebral blood flow and vascular perfusion, reduced resting pulse (which prevents microbleeds resulting from prolonged intense pulsatile stress on arteries), enhanced endothelial function and improved small vessel integrity [7, 71–73]. Several mechanisms have been proposed to underpin these protective effects, including increased endothelial progenitor cells and greater release and bioavailability of nitric oxide (a vasoactive substance essential for the vascular reactivity and the control of blood flow) by VEGF stimulation (as reviewed by [74]).

Conversely, lipid dysfunction, endothelial injury and vascular disease are risk factors for the development and progression of various types of dementia, including AD, where *APOE* ε4 carriage plays a major role. As described in the review on the vascular contribution to AD by Altman et al. (2010) [75], the conformation and lipidation state of ApoE isoforms affects their function, which includes the assembly, processing and removal of plasma lipoproteins. As they explain, lipoproteins assist with lipid transport and their normal functioning is key in the brain, given that lipids constitute a majority of its dry mass. ApoE plays a major role in the transportation and homeostasis of cholesterol in the brain, binding lipids primarily through interactions with the ATP-binding cassette transporter 1 (ABCA1), forming HDL-like particles. Additionally, ApoE4, unlike ApoE3, interacts with triglyceride-rich lipoproteins, causing linear conformational changes in ApoE that alter its binding properties. Across cell studies, it has been shown that ApoE4 reduces astrocytes’ ability to export cholesterol and mediates the reverse mechanism, the efflux of toxic peroxidated lipids from neurons to astrocytes for its clearance, which is key for neuroprotection at high levels of oxidative stress (as synthesised by [76]). ApoE4, presenting lower affinity for lipids compared to other ApoE isoforms, seems to also contribute to insufficient lipid availability for neuronal remodelling and repair processes. Altered synaptogenesis and neurogenesis due to the depletion of lipid rafts cause a disruption of neural communication (see [77]).

ApoE or its receptors are expressed in most cells participating in the formation, maintenance (e.g. astrocytes and endothelial cells) and interaction (e.g. macrophages and microglia) with the BBB. Wide evidence (including a study using bioengineered human vessels) supports that ApoE4 compromises the integrity of the BBB, inducing degeneration of brain capillary pericytes and producing increased leakiness and deficient Aβ clearance through the BBB [78, 79]. A leaky barrier makes the brain more susceptible to toxins and pathogens and increases the risk of neuronal dysfunction and neurodegeneration, including AD [77, 80]. Moreover, BBB leakiness leads to a progressive accumulation of fatty molecules and macrophages causing atherosclerotic cerebrovascular disease, contributing to neurodegenerative processes [75, 77] such as AD. In this line, in humans, CSF markers of BBB pericyte injury predict future cognitive decline only in *APOE* ε4 carriers [81]. Ultimately, these cascades of events alter the integrity of the BBB, dysregulate cerebral blood flow, impair brain repair mechanisms and increase the risk of cerebral amyloid angiopathy. The alterations of cerebral blood flow are of particular interest, since hypoperfusion is a well-established feature of the AD human brain. However, blood flow modifications in *APOE* ε4 carriers seem to be non-linear and age- and region-dependent, where hyperperfusion is observed in cognitively normal ε4 carriers as a compensatory mechanism to meet the metabolic demands of hyperactive neuronal activity [79, 80, 82].

**Fig. 3:**
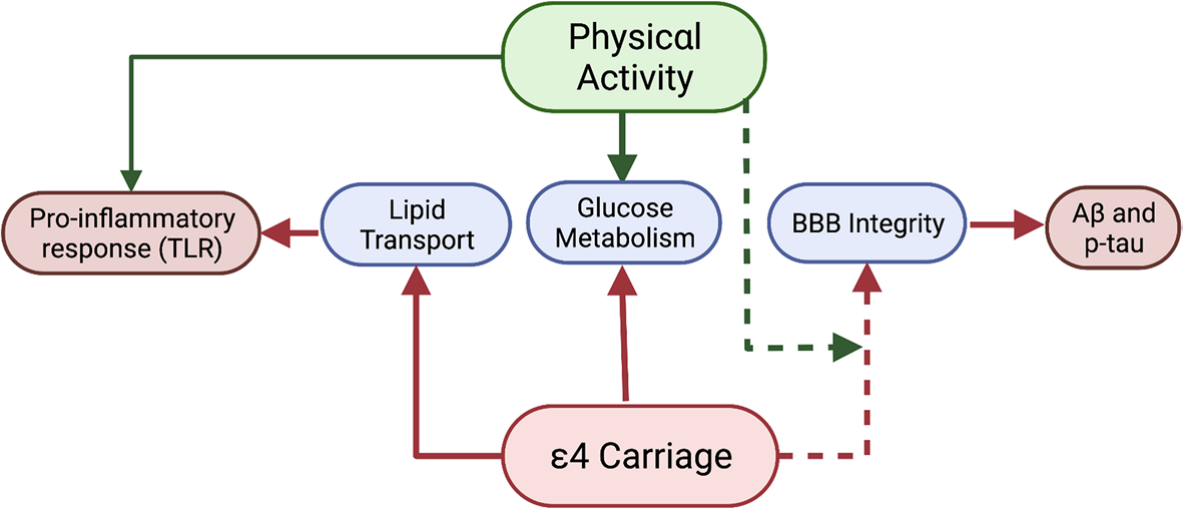
Hypothesised associations between apolipoprotein E, physical activity and cerebrovascular factors. Solid lines indicate empirically supported pathways, and dashed lines indicate hypothesised pathways with preliminary evidence. Green lines indicate a positive effect, and red lines indicate a negative effect. Abbreviations: Aβ amyloid-beta, *APOE* apolipoprotein E, p-tau phosphorylated tau, TLR toll-like receptor. Created by KS with BioRender.com

There is evidence that cerebrovascular adaptations following increased levels of PA might restore some, but not all, of the functions which are negatively affected by *APOE* ε4 carriage. For example, animal models show exercise prevents age-related decline in the integrity and function of the neurovascular unit in the frontoparietal cortex and the hippocampus, including greater preservation and coverage of pericytes [83]. However, most of these positive effects were lost in ApoE-deficient (ApoE − / −) mice. In wild type mice, ApoE expression decreases with age, but can be preserved with exercise engagement. Like *APOE* knockout mice, *APOE* ε4 transgenic mice and human carriers also show lower brain levels of ApoE. Therefore, it seems that PA might not be sufficient to preserve neurovascular health in *APOE* ε4 carriers. Alternatively, greater levels of PA engagement than those registered by Soto et al. (2015) [83] might be required for carriers to show benefits. In this vein, ApoE − / − mice under a high-cholesterol diet (an animal model of advanced atherosclerosis) did not show any benefits from PA, including no protective effects on BBB integrity [84]. Still, in humans, midlife PA has been shown to specifically reduce the risk of vascular dementia, independently of the *APOE* genotype [85]. Moreover, lower cerebral blood flow has been associated with higher physical fitness levels in a sample of healthy individuals where *APOE* ε4 carriers were reportedly over-represented, meaning that PA might be able to prevent the need for the activation of a potentially compensating mechanism [72]. In fact, healthy *APOE* ε4 carriers show higher cerebral blood flow than non-carriers in the hippocampus as a function of longer sedentary time [86]. Figure 3 presents a summary of hypothesised associations between *APOE* gene allele carriage, physical activity and cerebrovascular risk factors.

### Neuroimmune response

Bouts of PA are associated with a transient increase in anti- and pro-inflammatory cytokines, such as IL-1, IL-10, IL-18, IL-1 receptor antagonist (IL-1ra), IL-6 and C-reactive protein. However, while pro-inflammatory substances are released after exercising, physically fit individuals exhibit lower basal levels in comparison to their unfit and overweight counterparts, the latter of which tend to show a chronic state of low-level inflammation [87]. As a result of the expansion of adipose tissue, the level of pro-inflammatory adipokines (e.g. TNF, IL-6, IL-18) increases, while the level of anti-inflammatory cytokines decreases. According to a review, exercise favours a reduction in abdominal and visceral fat, thus reducing the release of pro-inflammatory substances and contributing to the increase in anti-inflammatory ones [88]. PA also contributes to improved immune function through elevating levels of myokines, including IL-6 (one of the most effective immune regulators), proportional to exercise duration and intensity. The immunomodulatory role of IL-6 stems from its ability to stimulate the release of IL-10 and IL-1ra and downregulate the release of TNF, promoting an anti-inflammatory state [89, 90]. Physical inactivity, systemic inflammation and age-related diseases are associated with an upregulation of toll-like receptors (TLRs), which have a key role in inflammation regulation through inducing the release of pro-inflammatory substances. Several review articles conclude that PA seems to reduce the expression of these receptors (specifically, TLR2 and TLR4) both after acute and regular exercise bouts [91, 92]. Finally, regular exercise contributes to lower baseline levels of pro-inflammatory monocytes and increased levels of circulating T regulatory cells [91, 93, 94].

The *APOE* genotype can modulate the innate immune response after an inflammatory stimulus, in animal models and humans, in vitro and in vivo. Specifically, ε4 allele carriage has been associated with increased immune reactivity. The ApoE4 protein is linked with a greater increment in the number of microglia, astrocytes and infiltrating T-cells and enhanced secretion and longer-lasting elevations of cytokines such as IL-1B, TNF-a and NO [95–98]. Additionally, *APOE* ε4 mice show basal structural and functional brain differences, including activated morphology of the microglia even in the absence of an inflammatory stimulus [96]. Zhu et al. [98] found lower levels of PSD95 and debrin in *APOE* ε4 homozygous mice compared to *APOE* ε3 homozygous mice, which might be indicative of differences in basal postsynaptic densities across genotypes. These differences might arise from chronic inflammation, which could make the brain more susceptible to damage accumulation across time [98]. Consequently, ApoE might behave as an anti-inflammatory agent, for example, the ApoE4 protein may be less efficacious than ApoE3 and ApoE2 at blocking inflammation [96, 98]. Others, however, suggest that ApoE4 may promote neuroinflammation and neurodegeneration [97]. In any case, the *APOE* ε4 allele has been consistently reported to increase the susceptibility to inflammation in a dose-dependent manner [95, 96].

The mechanisms through which ApoE4 contributes to the enhancement of inflammation are still being elucidated (see Fig. 1). Impairment or delay in the shift to the macrophage-orchestrated repair programme could be one contributing factor [96]. ApoE4 shows a diminished ability to induce a cholesterol efflux from lipid rafts in comparison to ApoE3, which might result in a greater activation of TLRs, leading to higher levels of inflammatory cytokines [95]. Another proposed mechanism to explain the immunological influence of ApoE variants is through TREM2 binding, which may be key for microglia activation and interaction with Aβ plaques [99]. It has been suggested that ApoE4 could be linked to higher microglial cell reactivity around Aβ plaques, compared to other isoforms, which may explain differences in plaque deposition [99]. Finally, ApoE variants reduce the classical complement cascade (CCC) activation by binding to C1q, forming a complex found in Aβ plaques in both animal models and human brains [100]. However, further research is needed to determine whether ApoE isoforms differentially reduce CCC activation, partially explaining the differential inflammatory responses evoked by each isoform.

Growing evidence suggests that the detrimental effects of the *APOE* ε4 allele carriage on cognitive performance, Aβ deposition and dementia risk could be mitigated or compensated by regular, moderate levels of PA (as reviewed by [101, 102]). Still, there is a striking lack of empirical evidence regarding the impact of the *APOE* ε4 genotype*PA interaction on the brain immune response. This is a promising field of research given that both ApoE and PA independently modulate key players of the immune system (e.g. IL-6, IL-10, TLRs).

### Brain glucose metabolism

Cerebral glucose hypometabolism is commonly observed in AD, which has been referred to as brain-specific “diabetes mellitus type 3” [103]. In a recent *JAMA* perspective publication, glucose metabolism impairments have been suggested to trigger vascular dysfunction in the brain, and such impairments are considered a modifiable causal factor, rather than a symptom of AD [104]. In fact, there is epidemiological evidence that diabetes mellitus type 2 patients are at a higher risk of developing AD and that effective treatment can reduce this risk [105].

Exercise can elicit a series of adaptations improving insulin signalling, glucose transport (mostly through GLUT4 translocation) and glucose metabolism in muscles (as reviewed by [106, 107]). Several recent reviews conclude that exercise engagement can improve peripheral insulin sensitivity both acutely and chronically, in insulin-resistant patients and healthy individuals [106, 108]. There is little research investigating insulin resistance and glucose metabolism within the CNS; however, initial results are promising. For example, increases in cardiorespiratory fitness (not mere increases in PA engagement) after an exercise intervention in humans resulted in improved brain glucose metabolism [109]. Animal models show that PA can reduce insulin resistance both in the periphery and in the brain [110, 111]. Exercise can also enhance mitochondrial function in the hippocampus of mice with obesity-induced insulin resistance [112].

On the other hand, mouse studies suggest that ApoE4 may impair insulin signalling and insulin-mediated mitochondrial respiration and glycolysis [113]. Among memory-impaired older adults, only *APOE* ε4 non-carriers seem to benefit from nasal insulin administration in terms of improved memory performance [114]. Cerebral glucose hypometabolism is a well-established marker of AD, which is exacerbated in *APOE* ε4 carriers in a region-specific [115, 116] and dose-dependent manner, compared to non-carriers [117]. Furthermore, regional glucose metabolism has been identified as a risk factor for MCI in cognitively normal older adults [118] and glucose metabolism declines faster among *APOE* ε4 carrier MCI patients [119]. Neurons have high energetic demands, relying heavily on glucose availability, which is mediated by glucose transporters. Reduced glycolytic flux and lower concentrations of glucose transporters (particularly GLUT3, the predominant brain isoform) are associated with AD severity in humans [120]. Relevantly, lower levels of insulin receptors and transporters have been found in the brain of *APOE* ε4 gene-targeted replacement (TR) mice and in *APOE* ε4 carrier AD patients compared to non-carriers, indicating hindered neuronal glucose uptake [121]. *APOE* ε4 TR mice show lower levels of glucose transporters (mostly GLUT3), synthesise less hexokinase (an enzyme involved in glycolysis) and produce lower glycolytic outcomes as they age, leading to less efficient energy production in brain cells [122, 123]. In mice, ApoE4 has also been linked to multiple markers of mitochondrial dysfunction, including lower protein levels of complexes I–V, reduced mitochondrial oxidative phosphorylation and energy metabolism and decreased ATP synthesis [124].

Unfortunately, there is a lack of empirical evidence on the combined effect of *APOE* ε4 carriage and PA on cerebral glucose metabolism. However, available data on each independent mechanism suggest that PA might counteract the detrimental effects of genetic risk.

## The combined effect of physical activity and *APOE* ε4 carriage on AD-related mechanisms: an integrative model proposal

**Fig. 4:**
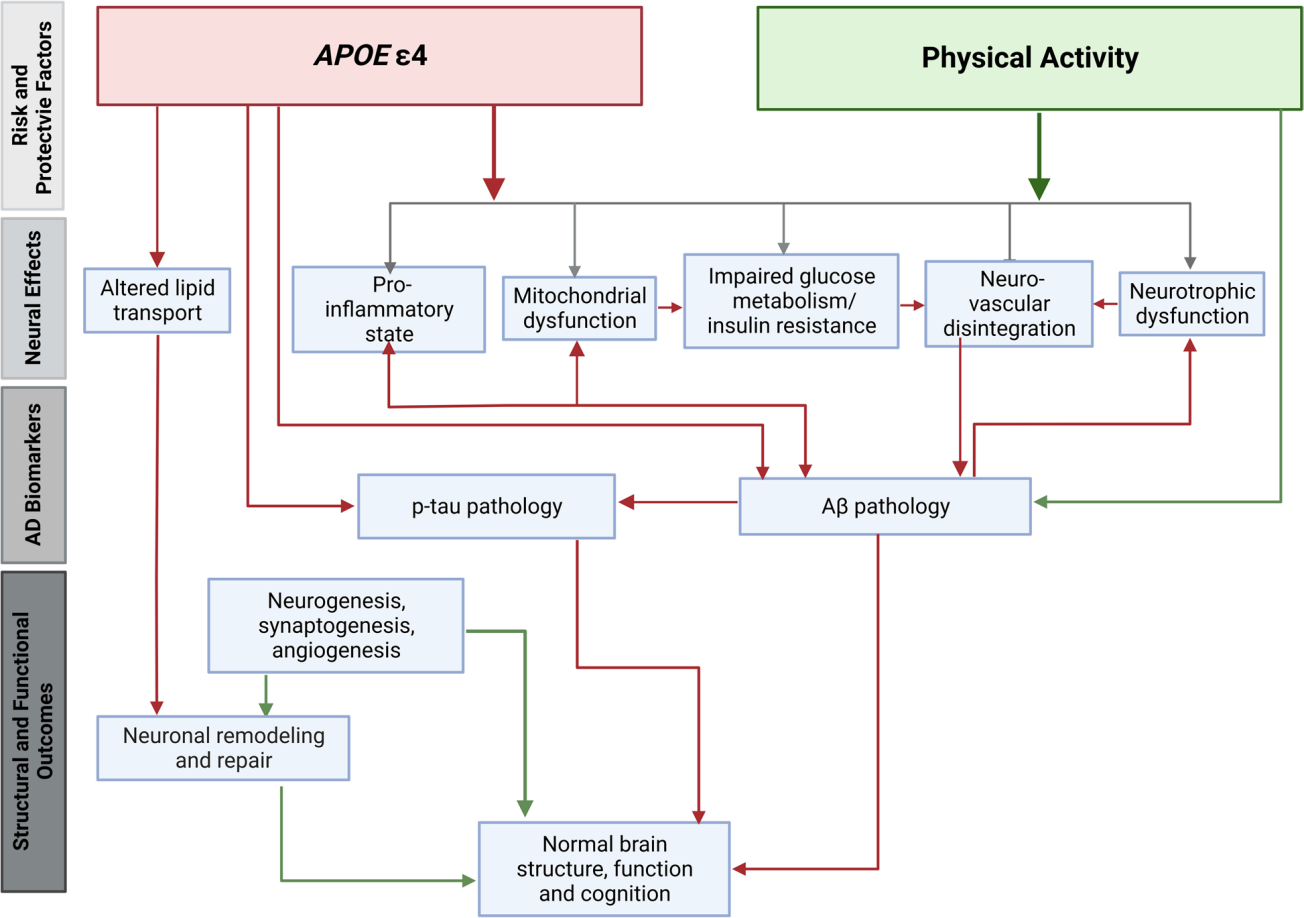
An integrative model of hypothesised associations between apolipoprotein E ε4 carriage, physical activity and Alzheimer’s disease mechanisms. Green lines indicate a positive effect, and red lines indicate a negative effect. Abbreviations: Aβ amyloid-beta, *APOE* apolipoprotein E, p-tau phosphorylated tau. Created by KS with BioRender.com

In this section, we aim to propose a comprehensive overview of how the PA*APOE interaction is associated with various mechanisms involved in AD pathology (as discussed above). Where there is evidence to suggest that multiple outcomes may be influenced by PA and *APOE* in a bi-directional or synergistic manner, they have been included in the same model (see Fig. 4). This narrative also emphasises the speculative associations that remain to be fully elucidated, providing testable hypotheses for future studies (see Table 1).

**Table 1:** Suggestions for prospective studies. Abbreviations: *Aβ* amyloid-beta, *APOE* apolipoprotein E—gene, ApoE apolipoprotein E—protein, *BBB* brain-blood barrier, *CNS* central nervous system, *PA* physical activity, *p-tau* phosphorylated tau, *TLR* toll-like receptor

**Identified testable hypotheses**
1. In *APOE* ε4 carriers, PA, via downregulation of pro-inflammatory cytokines and preservation of the BBB, will reduce the negative effects of pro-inflammatory molecules on Aβ burden 2. In *APOE* ε4 carriers, PA, via downregulation of pro-inflammatory cytokines and preservation of the BBB, will reduce the negative effects of pro-inflammatory molecules on tau hyperphosphorylation 3. PA-induced Aβ clearance by means of protease upregulation will be more efficient in *APOE* ε4 non-carriers 4. Immunosenescence and age-related downregulation of neurotrophic factors reduce the ability of PA to counteract the detrimental effects of *APOE* ε4 carriage 5. PA will less efficiently contribute to the preservation of capillary pericytes, small vessels and the BBB in the presence of the *APOE* ε4 allele 6. Alternatively, greater levels of PA might be required for *APOE* ε4 carriers to show improvements in the integrity of the BBB 7. PA will improve insulin signalling and glucose metabolism in the CNS even in the presence of the *APOE* ε4 allele 8. Midlife PA will reduce the risk of brain insulin resistance in late life, even in the presence of the *APOE* ε4 allele
**Relevant exploratory questions**
1. Can PA counteract the increased Aβ oligomerization driven by ApoE4 presence? 2. Does PA, through increased expression of neurotrophic factors, still promote neurogenesis and synaptogenesis despite the detrimental effects of *APOE* ε4 carriage on lipid transport? 3. How does *APOE* genotype modulate the neurotrophic response to PA in clinical subgroups at various stages of disease progression? 4. Does PA improve glucose metabolism, glucose transporter levels, insulin receptor and transporter levels and mitochondrial function in the presence of the *APOE* ε4 allele? 5. Can PA revert brain insulin signalling and glucose metabolism impairments regardless of *APOE* genotype?

Aβ oligomer neurotoxicity and aggregation is the most studied marker of AD, and both PA and *APOE* ε4 carriage influence Aβ production and clearance in opposing directions. In fact, ε4 carriers might benefit the most from PA, at least in terms of reduced Aβ deposition [12]. Whether PA also counteracts *APOE* ε4 carriage (associated to enhanced Aβ oligomerization [16]) in terms of decreased soluble Aβ remains to be understood. This is important, given that it is widely accepted that the neurotoxicity associated to Aβ oligomers rather than Aβ plaques drives most of the detrimental downstream effects leading to AD neuropathology [15]. For example, both in transgenic mice and in AD patients in a phase 2 randomised clinical trial, pharmaceutical agents targeting Aβ only show clinical efficacy when they are directed towards Aβ oligomers, but not monomers or plaques [125]. In this line, Aβ oligomers have been identified to cause mitochondrial dysfunction, and so does the presence of ApoE4 [124]. PA seems to improve mitochondrial function [112], but its effects in *APOE* ε4 carriers remain unknown.

Another defining feature of AD is tau pathology, although research on how PA and/or *APOE* ε4 carriage modulate tau is very scarce. Preventing tau pathology is crucial, since this biomarker is more closely associated with neurodegeneration and cognitive decline than amyloid pathology alone [126, 127]. Tau pathology may be exacerbated in ε4 carriers, potentially mediated by the presence of Aβ oligomers [41, 42]. In this case, PA, through its positive effects on Aβ (in the presence of the *APOE* ε4 allele), might prevent increased tau pathology. This hypothesis could be examined by longitudinal examinations of brain Aβ and tau in relation to physical activity levels, with examination of the moderating effects of *APOE* ε4 allele carriage.

A vast body of evidence shows that basal levels of pro-inflammatory molecules are higher among *APOE* ε4 carriers [95, 96] and lower in physically active individuals [91]. Surprisingly, to the best of our knowledge, the interaction between these two factors regarding inflammation is yet to be investigated. There appears to be a bi-directional relationship between amyloid pathology and pro-inflammatory molecules (both directly influenced by PA and *APOE* ε4 carriage with opposing effects), in that increased inflammatory cytokines may lead to greater Aβ, but also the neurotoxic environment induced by Aβ may lead to greater inflammation [128–130]. Animal models also show that exercise interventions can modulate levels of pro-inflammatory cytokines, which coincides with a reduction in Aβ levels [131, 132]. Meanwhile, it remains unclear whether modulating inflammatory markers is a mechanism through which exercise reduces Aβ, or whether exercise influences inflammation and Aβ through independent pathways. In a similar fashion, the release of pro-inflammatory substances has been linked to an increase in tau phosphorylation [40], providing another pathway through which PA could contribute to preventing or ameliorating AD pathology. Therefore, it is fundamental to understand the extent of the ability of PA to regulate the immune response even in the presence of the *APOE* ε4 allele.

Probably the most investigated mechanism through which PA exerts its beneficial effects on brain health is the upregulation of neurotrophic factors [56, 58, 59], particularly in the hippocampus. These molecules include BDNF, IGF-1 and VEGF, and they promote neurogenesis, synaptogenesis and angiogenesis. The enhancement of neurotrophic response might compensate, at least temporarily, for the detrimental effects of AD pathology on brain structure and function. The expression of these neurotrophic factors seems to be reduced in the presence of the *APOE* ε4 allele [61, 65, 66], indicating the *APOE* ε4 allele might moderate the acute neurotrophic response to a bout of PA, a hypothesis that requires further investigation. Still, it appears that in *APOE* ε4 carriers neurotrophic function (i.e. BDNF and TrkB levels) is partially restored in response to habitual PA [68]. Future studies should examine the moderating role of the *APOE* ε4 allele on the PA-induced changes to other neurotrophins and their impact on AD pathology. For example, it seems plausible that PA-induced VEGF could potentiate neuroprotection and prevent Aβ and p-tau aggregation. Additionally, some of these associations may be bi-directional, as aggregation of Aβ plaques can also downregulate BDNF and IGF-1 expression [133], although no study to date has investigated these interactions in the context of PA research. There is also evidence that the upregulation of pro-inflammatory cytokines in ε4 carriers may influence neurotrophic expression, specifically VEGF [70]. However, there is currently limited evidence that *APOE* isoform-dependent neurotrophic expression is influenced by inflammatory factors, and further research is required.

Some of the most pernicious outcomes associated with *APOE* ε4 carriage result from the alteration of lipid transport in the brain, which ultimately deteriorates neural remodelling and repair mechanisms, disrupting neuronal communication [77]. Accordingly, it would be beneficial to examine whether neurogenesis and synaptogenesis are negatively affected by the carriage of *APOE* ε4, even when the neurotrophic response to exercise is preserved. Furthermore, lipid transportation impairment has been associated with greater pro-inflammatory responses, via TLR activation, which would lead to the cascade of effects explained above [95, 99]. Given the fact that PA downregulates the expression of TLRs [91, 92], increasing habitual PA might be an effective intervention to prevent some of the ε4 carriage-related noxious effects.

ApoE4 is exceedingly detrimental to the integrity of the BBB, which undermines Aβ clearance, making the brain more susceptible to toxins [78, 79, 134]. In this case, unfortunately, PA has not been found to show a protective effect on the neurovascular unit in the presence of the *APOE* ε4 in animal models [83]. However, it remains plausible that greater levels of PA are required for ε4 carriers to preserve BBB integrity. Promisingly, human studies show that PA exerts a positive effect on cerebrovascular health in *APOE* ε4 carriers [86]. PA-induced increases in VEGF expression could mediate these beneficial outcomes. Finally, vascular integrity also seems to be compromised when glucose metabolism is impaired [104], and *APOE* ε4 carriage negatively impacts glucose metabolism in the brain. Conversely, PA improves insulin signalling, glucose transport and glucose metabolism [106, 107]. These effects have been mostly observed peripherally, although animal studies provide favourable results within the CNS [110–112]. However, the combined effect of PA and *APOE* ε4 carriage on brain glucose metabolism has not been addressed to date. Still, given that both factors act on the same pathways, PA could become a strategic tool to prevent insulin resistance and vascular dysfunction in ε4 carriers, two features that characterise AD neuropathology.

In brief, *APOE* ε4 carriage promotes AD pathology through multiple pathways, where its effects on cerebrovascular health and Aβ pathology are particularly detrimental. Although Aβ deposition has not been identified as a main contributor to clinical symptomatology in AD, Aβ oligomerization exerts a series of downstream effects that trigger additional pathological effects (e.g. the exacerbation of tau pathology). Moreover, Aβ oligomers, directly and indirectly, seem to impede the capacity of PA-induced protective mechanisms to operate. In this line, the neurotrophic response to PA is likely the most powerful tool to compensate for AD pathology in a clinically relevant manner. Yet, neuroinflammation and altered lipid transportation as a result of *APOE* ε4 carriage and/or Aβ oligomerization hinder PA-induced neurogenesis, synaptogenesis and angiogenesis as well as neuronal and vascular repair. Therefore, it urges to investigate:

- In early life and at the preclinical stages of the pathology, whether PA can reduce the first hallmarks of the disease (including Aβ oligomerization).
- At more advanced stages, whether specific PA regimes (e.g. exercise at higher intensity) can overcome the above-listed impairments of PA-induced protective mechanisms or whether pharmaceutical agents improving lipid transportation or ameliorating neuroinflammation can be combined with PA to achieve the desired outcomes.

This and other key questions for future research have been included in Table 1.

## Major challenges

This theoretical article represents the first attempt to create a comprehensive framework to integrate the complex relationships that occur between *APOE* ε4 carriage and PA engagement at multiple levels, which ultimately modulate AD risk. From our perspective, at least part of the variability within this field emerges as a result of the usage of different outcome measures to characterise the interaction between these two contributing factors. For instance, as we have reported here, the detrimental effect of *APOE* ε4 carriage on Aβ pathology seems to be counteracted by PA engagement, at least at the early stages of the disease. However, PA does not seem to be protective enough against the damage *APOE* ε4 carriage causes to the BBB, which eventually might lead to additional downstream impairments, including an exacerbation of Aβ pathology. AD is a complex disease and the multiple mechanisms involved in AD pathogenesis are interrelated [135–138], where alterations in certain pathways might trigger further disturbances on other pathways. Besides the mechanistic complexity of AD, this disease is also characterised by a prolongated pathological process, which is believed to last up to 50 years from the earliest silent molecular alterations to the patient’s decease. Along the AD continuum, certain effects might be non-linear and time dependent. For example, the neurotrophic response to PA may be contingent on the degree of AD-related neuropathology present, particularly as a more advanced aggregation of Aβ plaques can independently downregulate BDNF and IGF expression [133]. Due to the nascent stage of this research area, it is not yet possible to form a single evidence-based model which includes *all* relevant associations between the AD-related outcomes discussed above. Therefore, in addition to our proposed integrative model, we also suggest directions for future research to allow the development of such a comprehensive model (see Table 1).

There are key methodological considerations that should be carefully addressed when designing prospective studies. Firstly, methodological variations in biomarkers assessment hinder comparisons across studies. Moreover, there is currently a critical lack of longitudinal studies examining both PA and *APOE* genotype looking at the evolution of biomarker levels rather than AD incidence in humans. This obstacle has been partially overcome in animal studies. Moreover, animal studies enable the investigation of certain research questions that nowadays would not be possible to address in humans (e.g. the acute effect of exercise on hippocampal expression of TrkB). However, there are important differences in the *APOE* gene between mice and humans, complicating research on the field [139, 140]. Most animal studies have utilised transgenic mice carrying the human *APOE* ε4 variant, where they often induce the expression of classical AD biomarkers, such as Aβ. Finally, in human studies, PA levels and exercise routines are often poorly measured and described, while animal studies impose certain limitations on the PA and exercise regimes that can be tested (e.g. strength/balance training programmes, daily activity contribution to overall PA). More research is needed to establish the PA parameters (i.e. type, volume, intensity and frequency) that maximise beneficial outcomes on specific mechanisms in at-risk populations and at different disease stages. These much-needed pieces of information are key to upgrade the model proposed.

## Conclusions

Through this theoretical article, we aim to propose a novel integrative model of how PA and *APOE* ε4 carriage, independently and in combination, influence the pathogenesis of AD. Looking at the reviewed mechanisms, we identify potential pathways through which the beneficial effects of PA might offset some of the detrimental outcomes of *APOE* ε4 carriage. Nonetheless, PA does not seem to be able to entirely prevent or revert the noxious effects of genetic risk. This is in line with the idea that PA delays rather than prevents AD neuropathology. Accordingly, the potential of PA to exert its benefits might be dependent on the disease stage and the extent of the damage to the above-reviewed mechanisms. Still, given that the molecular alterations associated with AD remain silent for up to 20 years, delays in the onset and progression of neuropathological changes could be clinically meaningful. Moreover, compared to pharmaceutical approaches to AD, which usually tackle one specific pathway, PA influences a wide array of molecular targets that all lie somewhere in the stream of AD risk. Therefore, although PA may not be sufficient to prevent or cure AD, it represents a strong complementary therapeutic tool to be combined with more precise pharmacological interventions.

## Data Availability

Not applicable.

## Abbreviations

Aβ: Amyloid-beta
AD: Alzheimer’s disease
ABCA1: ATP-binding cassette transporter 1
ADAM-10: A disintegrin and metalloproteinase domain-containing protein 10
*APOE*: Apolipoprotein E—gene
ApoE: Apolipoprotein E—protein
APP: Amyloid precursor protein
BACE: β-Site amyloid precursor protein cleaving enzyme 1
BBB: Blood-brain barrier
BDNF: Brain-derived neurotrophic factor
CCC: Classical complement cascade
CDK5: Cyclin-dependent kinase 5
CNS: Central nervous system
CSF: Cerebrospinal fluid
GLUT: Glucose transporter
GSK3: Glycogen synthase kinase 3
HDL: High-density lipoproteins
IDE: Insulin-degrading enzyme
IGF: Insulin-like growth factor
IL: Interleukin
ISF: Interstitial fluid
LDL: Low-density lipoprotein
LDLR: LDL receptor
LRP1: LDL receptor–related protein 1
LTP: Long-term potentiation
MCI: Mild cognitive impairment
NFT: Neurofibrillary tangle
NO: Nitric oxide
PA: Physical activity
PS1: Presenilin
p-tau: Phosphorylated tau
TLR: Toll-like receptor
TNF: Tumour necrosis factor
TR: Targeted replacement
TREM2: Triggering receptor expressed on myeloid cells 2
TrkB: Tropomyosin receptor kinase B
VEGF: Vascular endothelial growth factor
VLDL: Very low-density lipoprotein
VLDLR: VLDL receptor
